# Shelf Life Extension of Chilled Pork by Optimal Ultrasonicated Ceylon Spinach (*Basella alba*) Extracts: Physicochemical and Microbial Properties

**DOI:** 10.3390/foods10061241

**Published:** 2021-05-29

**Authors:** Yuthana Phimolsiripol, Srirana Buadoktoom, Pimporn Leelapornpisid, Kittisak Jantanasakulwong, Phisit Seesuriyachan, Thanongsak Chaiyaso, Noppol Leksawasdi, Pornchai Rachtanapun, Nareekan Chaiwong, Sarana Rose Sommano, Charles S. Brennan, Joe M. Regenstein

**Affiliations:** 1 Faculty of Agro-Industry, Chiang Mai University, Chiang Mai 50100, Thailand; s.buadoktoom@gmail.com (S.B.); kittisak.jan@cmu.ac.th (K.J.); phisit.seesuriyachan@gmail.com (P.S.); thanongsak.c@cmu.ac.th (T.C.); noppol@hotmail.com (N.L.); pornchai.r@cmu.ac.th (P.R.); meen.nareekan@gmail.com (N.C.); 2 Cluster of Agro Bio-Circular-Green Industry, Chiang Mai University, Chiang Mai 50100, Thailand; sarana.s@cmu.ac.th; 3 Faculty of Pharmacy, Chiang Mai University, Chiang Mai 50200, Thailand; pim_leela@hotmail.com; 4 Faculty of Agriculture, Chiang Mai University, Chiang Mai 50200, Thailand; 5 School of Science, STEM College, RMIT University, Melbourne 3000, Australia; charles.brennan@rmit.edu.au; 6 Department of Food Science, Cornell University, Ithaca, NY 14853-7201, USA; jmr9@cornell.edu

**Keywords:** Ceylon spinach, *Basella alba*, ultrasonication, antioxidant, antibacterial activity, pork, *Sus scrofa*

## Abstract

The effect of ultrasonication on the antioxidant and antibacterial properties of Ceylon spinach (*Basella alba*) extracts (CE) and the shelf life of chilled pork with CE were studied. The CE were ultrasonicated at different power levels (60–100%) for 10–40 min in an ultrasonic bath with the rise of antioxidant activities (*p* ≤ 0.05) proportional to the ultrasonication time. The additional investigation of antibacterial activities showed that the ultrasonicated extracts (100 mg/mL) could inhibit and inactivate *Staphylococcus aureus* and *Escherichia coli* with the optimal condition of 80% power for 40 min. For shelf life testing, fresh pork treated with the ultrasonicated extracts at 100 and 120 mg/mL had lower values of thiobarbituric acid reactive substances (TBARS) than the control (without dipping). For food safety as measured by the total microbial count, the fresh pork dipped with 100–120 mg/mL CE extract could be kept at 0 °C for 7 days, 2 to 3 days longer than control meat at 0 and 4 °C, respectively. A sensory evaluation using a nine-point hedonic scale showed that fresh pork dipped with 100-mg/mL CE extracts was accepted by consumers. It is suggested that CE extracts can be applied in the food industry to enhance the quality and extend the shelf life of meat products.

## 1. Introduction

Pig meat (pork) is one of the most eaten meats in the world, and pork is a human food cooked or processed. The Food and Agriculture Organization (FAO) forecasted global pork production, the direction of pork production is predicted to rise to 131 million tons in 2028 from 121 million tons in 2018 [1]. The meat industry is focused on consumer awareness of meat production for food safety to prevent foodborne diseases, and microbial growth can lead to food spoilage [2]. Antioxidants have been applied in meat and meat products to reduce oxidation [3]. The interaction of natural antibacterial-active extracts and packaging or storage methods appears to be the most economically appropriate technology known as bio-preservation strategies [4,5]. Moreover, lipid and protein oxidation cause the loss of meat quality and a shorter shelf life. Lipid oxidation can produce effects in meat by changing the sensory properties [6]. In addition, de Souza de Azevedo et al. [7] also applied nisin by dipping or spraying for the shelf life extension of pork meat. The utilization of plant extracts as alternatives for meat preservation, including burgers during storage, will be beneficial for both the industry and consumers [8].

Ultrasonication is a green extraction technology that is cost-effective, adaptable, efficient, and effective for extracting natural food ingredients [9]. The extraction times and high temperatures can be mitigated with improved yields ensuring the preservation of the active ingredients [10]. Ultrasound-assisted extracts have been shown to possess greater antioxidant and antimicrobial properties than conventional extraction samples [11]. The acoustic cavitation of an ultrasound facilitates the cell permeability of solvents through damaged cells walls [12]. For example, Thai propolis, which was extracted using an ultrasound for 30 and 60 min, showed increased antibacterial activities against *Micrococcus luteus*, *Listeria monocytogenes*, and *Escherichia coli* [13], with the lowest IC_50_ (50% inhibitory concentration) for the scavenging DPPH radicals when ultrasonicated for 15 min. The ultrasonication method could also produce extracts with higher antibacterial activities against *S. aureus* and *Bacillus subtilis* than the extraction using maceration [14]. Moreover, combined Soxhlet and ultrasonication has also been used for oleaginous seed extraction, which can improve the conventional Soxhlet extraction, resulting in higher yields and shorter extraction times [15].

Ceylon spinach (*Basella alba*) is a popular local vegetable in Thailand rich in vitamins A and C, phenolic compounds, and several other antioxidants. It is low in calories (by volume) and high in protein [16]. *Basella alba* has a long history of use as an additive for food preservation [17], as well as medicinal compounds that are used in astringents, demulcents, laxatives, and soothing agents [18]. Kumar et al. [19] reported in vitro assays in preclinical and clinical studies that have shown that *Basella* has antibacterial, antihyperglycemic, anti-inflammatory, and antiproliferative activity and is cytotoxic. Maran et al. [20] investigated the extraction of *Basella rubra* L. pigments using an ultrasound. They confirmed that an extraction with 94-W ultrasound power at 54 °C for 32 min with a solid:liquid ratio of 1:17 g/mL resulted in the maximum yield of betacyanin (1.43 mg/g) and betaxanthin (5.37 mg/g). Furthermore, Adesina et al. [21] found that the levels of the phospholipids of *Basella alba* and *Basella rubra*, using a Soxhlet extraction, were 1680 and 1920 mg/100 g, respectively. However, the antioxidant and antibacterial activities of Ceylon spinach extracts using Soxhlet combined with ultrasonication and its application in the shelf life of pork have not been investigated. Therefore, the objectives of this study were to investigate the effects of ultrasonic power and time on the antioxidant and antibacterial properties of *Basella alba* stems and to investigate the shelf life extensions of chilled, fresh pork mixed with stem extracts.

## 2. Materials and Methods

### 2.1. Plant Materials

Fresh Ceylon spinach (*Basella alba*) stems at 1.5–2 months after planting were collected from the College of Agriculture and Technology field plots (Mueang, Chiang Mai, Thailand) during February 2019. The samples were washed with tap water and dried in a hot-air oven (UNB 400, Memmert, Eagles, WI, USA) at 50 °C for 24 h. The dried plants were ground using an electric grinder (BL3071AD, Tefal, Bangkok, Thailand) and stored at −20 °C before extracting within 3 months.

### 2.2. Chemicals and Reagents

2,2-Diphenyl-1-picrylhydrazyl (DPPH), 2,2′-azino-bis (3-ethylbenzothiazoline-6-sulfonic acid (ABTS), 2,4,6-tris(2-pyridyl)-s-triazine (TPTZ), gallic acid (GA), and 2-thiobarbituric acid were purchased from Sigma–Aldrich (Singapore, Singapore). Ferrous sulfate, Na_2_CO_3_, and the Folin–Ciocalteu reagent were bought from Loba Chemie (Mumbai, India). Nutrient broth, Mueller–Hinton broth (MHB), and Mueller–Hinton agar (MHA) were purchased from Himedia (Mumbai, India). Plate count agar (PCA) and peptone water were purchased from Difco (Cockeysville, MD, USA). Other chemicals were analytical grade and obtained from RCI Labscan (Bangkok, Thailand).

### 2.3. Preparation of Extracts

**Figure 1:**
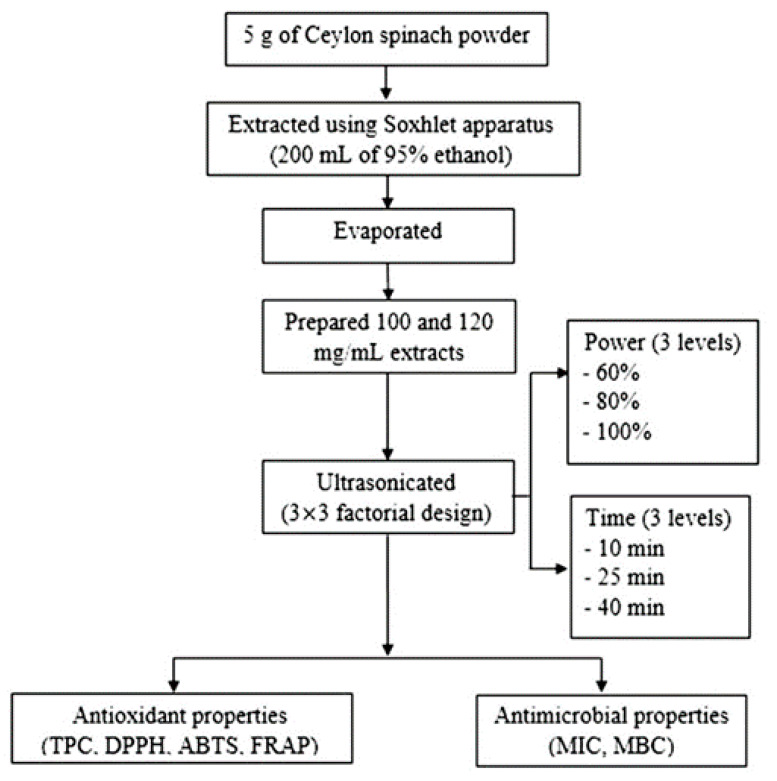
Design of the ultrasonic experiment.

Ceylon spinach powder (5 g) were extracted using a Soxhlet apparatus (Quicklet, Northern Ireland, UK) with 200 mL of 95% (*v*/*v*) ethanol at 80 °C for 4 h [16]. The extract was concentrated at 175-mbar reduced pressure in a water bath (B-490, Buchi, Saint Gallen, Switzerland) at 60 °C using a rotary evaporator (CH-9230, Buchi) to evaporate the ethanol, and then, the evaporated sample was further dried using a vacuum oven at 50 °C (VD53, Binder, Tuttlingen, Germany) to obtain dry Ceylon spinach extracts (CE) following the method of Sulaiman et al. [22]. The CE (100-mg of dry, crude extract after the evaporation/mL of distilled water), which corresponded to 1:10 (*w*/*v*), were ultrasonicated following the method adapted from Hashemi et al. [23]. The experiment had two factors: the extraction time (10–40 min) and power (60–100%) and were arranged as a 3 × 3 factorial in a completely randomized design (CRD) with duplicates, as shown in Figure 1. The CE samples in the test tube were ultrasonicated using an ultrasonic bath (40 kHz, 150 W, SB25-12DTD, Drawell, Jacksonville, FL, USA) at 25 °C. The untreated ultrasonication sample was used as the control.

### 2.4. Antioxidant Properties of Ultrasonicated CE

#### 2.4.1. Total Phenolic Compounds (TPC)

TPC were determined as described by Oluwakemi et al. [24]. Briefly, 500 µL of the sample (100 mg/mL) was mixed with 2.5 mL of 10% Folin–Ciocalteu reagent and 2 mL of 7.5% Na_2_CO_3_. After incubation in the dark at 30 °C for 30 min, the absorption was measured at 765 nm using a multi-mode microplate reader (SpectraMax^®^ i3x, Molecular Devices, San Jose, CA, USA). Aqueous solutions of GA were used to prepare the calibration curve. Results (*n* = 3) were expressed as GA equivalents (E).

#### 2.4.2. DPPH and ABTS Radical Scavenging Activity and Ferric-Reducing Antioxidant Power (FRAP)

Free radical scavenging activity was determined using the DPPH radical assay of Surin et al. [25]. The sample solution (2 mL) was mixed with 2 mL of 0.2-mmol/L DPPH solution. After incubation in the dark at 30 °C for 30 min, the absorbance was measured at 517 nm. The percentage of inhibition of the DPPH radical (*n* = 3) was calculated. The results were expressed as IC_50_ values, the lowest concentration of the sample required to inhibit 50% of the radicals.

For the ABTS radical scavenging assay of Chaiwong et al. [26], 100 µL of the sample from 0.5–5.0 mg/mL was mixed with 900 µL of 7-mM ABTS reagent. After incubation in the dark at 30 °C for 6 min, the absorbance was measured at 734 nm. The percentage of inhibition of the ABTS radical was calculated. The results (*n* = 3) were expressed as the IC_50_ values.

The reducing power was determined using the FRAP assay of Surin et al. [27], with some modifications. Briefly, 100 µL of the sample (100 mg/mL) was mixed with 1900 µL of FRAP reagent, which consisted of 2.5 mL of 10-mM TPTZ in 40-mM HCl, 2.5 mL of 20-mM FeCl_3_ and 25 mL of 0.3-M acetate buffer (pH 3.6). The absorption was measured at 595 nm, and aqueous FeSO_4_ solutions were used to prepare the calibration curve. The measurements were done in triplicate (*n* = 3). The untreated ultrasonication sample was used as the control.

### 2.5. Antibacterial Activities of Ultrasonicated CE Using the Minimum Inhibitory Concentration (MIC) and Minimum Bactericidal Concentration (MBC)

The MIC is the concentration of CE required to inhibit the growth of the tested microorganism. The ultrasonicated samples were prepared at 100 mg/mL of crude extract after evaporation, and serial dilutions were done to obtain solutions at 50, 25, 12.5, 6.25, and 3.125 mg/mL using the modified method of Kumar et al. [28]. The tested microorganisms—namely, *S. aureus* TISTR 2320, *E. coli* TISTR 527, *Salmonella* Typhimurium TISTR 1469, and *Pseudomonas aeruginosa* TISTR 2370—were obtained from the Thailand Institute of Scientific and Technological Research (TISTR, Bangkok, Thailand). The initial microbial samples were, at ~1 × 10^8^ CFU/mL, obtained by adjusting the turbidity to match a 0.5 McFarland standard. Each sample solution (250 µL) was diluted with 250 µL of sterile MHB. The solution was inoculated with 250 µL of microbial suspension and then incubated at 37 °C for 24 h. Changes in the turbidity were measured at 600 nm for comparison with the control.

The MBC is the lowest concentration of extract with the ability to inactivate the tested microorganisms [28]. Six different concentrations of ultrasonicated CE were tested by streaking on MHA plates that were then incubated at 37 °C for 24 h. The lowest concentration of the plant extract required to inactivate the test microorganism was designated as the MBC value.

### 2.6. Shelf-Life Evaluation of Fresh, Chilled Pork

#### 2.6.1. Preparation of Fresh, Chilled Pork

**Figure 2:**
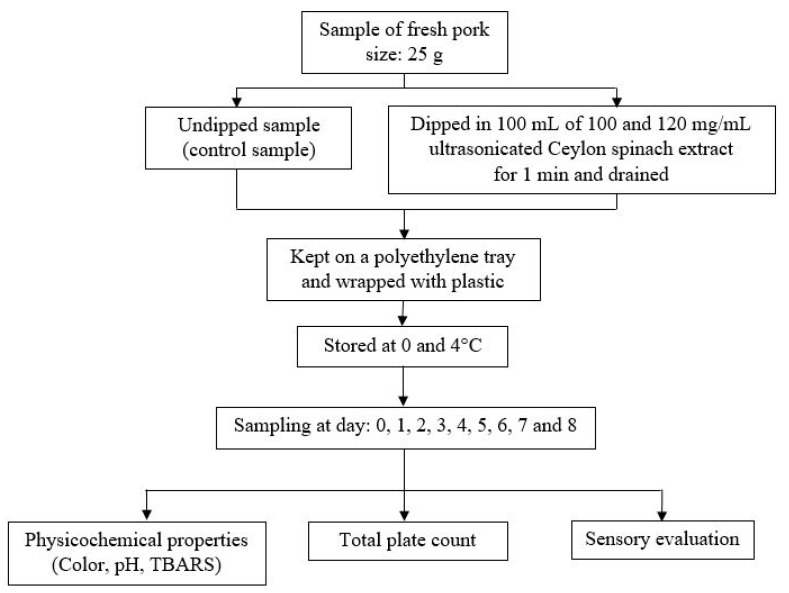
Design of the experiments for the shelf life study.

Fresh center-cut pork sirloins (Longissimus thoracis et lumborum, LTL) were selected and purchased from Charoen Pokphand Foods (Chiang Mai, Thailand) 1 day after slaughter and transported to the laboratory at 0–4 °C within 2 h. A Certificate of Analysis as a standard control for the fresh pork quality was provided by the company to ensure the safety of the product from the aerobic plate count, coliform bacteria, *E. coli*, *S. aureus*, *Clostridium perfringens*, *Salmonella* spp., *Enterococcus* spp., *L. monooxygenase*, *Campylobacter jejuni*, and yeast and molds following the standard US Food and Drug Administration protocols. The pork was sliced perpendicular to the long axis of the muscle with a knife into pieces of ~25 g with 2-cm thickness. Pork samples were dipped into two CE solutions with different concentrations (100 or 120 mg/mL) for 1 min. The preparation of the 100 or 120 mg/mL CE solution was done by dissolving 100 or 120 mg of dry ultrasonicated CE with 1 mL of distilled water. The optimum condition for ultrasonication was 80% power for 40 min, which was selected for the CE preparation. After dipping, the excess surface liquid on the samples were drained away and air-dried on a wire mesh at 20 °C for 10 min in a clean room before packing. The samples were kept in polyethylene (PE) trays (one/tray) and wrapped with food-grade polyvinyl chloride wrapping films (MMP, Bangkok, Thailand) with the experimental design shown in Figure 2. All samples were stored in two refrigerators (KB400, Binder, Bohemia, NY, USA) at 0 ± 1 and at 4 ± 1 °C and kept away from light for a period of 8 days and collected (10 pieces/treatment each time for different analyses) every day prior to comparison with the undipped control. Three pieces were used for the microbial analysis; another 3 pieces for the physicochemical analysis (color, pH, and TBARS); and the remaining 4 pieces were used for the sensory analysis.

#### 2.6.2. Color, pH, and TBARS Measurements

The color prior to blooming of the packed and chilled fresh pork after being unwrapped (*n* = 3), as mentioned by Sen et al. [29], was measured using the CIELAB system using a Chroma meter (CR-410, Konica-Minolta, Tokyo, Japan) and illuminant D65 observer angle of 2°; aperture size of 50 mm; and expressed as L*, a*, and b*. The lightness (L*) value indicates black at 0 and white at 100, the a* value represents the red (+60)–green (−60) color, and the b* value describes the yellow (+60)–blue (−60) color. A 25-g meat sample with 225-mL distilled water was homogenized with a hand blender (MSM64110, Bosch, Bangkok, Thailand) for 30 s at 30 °C before pH measurements (*n* = 3) using a pH meter (FiveEasy F20, Mettler Toledo, Greifensee, Switzerland). The pH meter was calibrated at 30 °C using pH buffers at 4.0 and 7.0 (RCI Labscan, Ltd., Bangkok, Thailand). The TBARS in chilled pork was determined using the method of Lekjing and Venkatachalam [30]. The meat (1 g) was homogenized using a Vortex mixer (VTX-3000L, LMS, Tokyo, Japan) in a 10-mL mixture of 0.375% (*w*/*v*) thiobarbituric acid, 15% (*w*/*v*) trichloroacetic acid, and 0.875% (*w*/*v*) of 0.25-M HCl. The mixture was heated at 100 °C for 10 min to develop a pink color and cooled down with tap water. The mixture was centrifuged (Rotina 380R, Hettich, Tuttlingen, Germany) at 1520× *g* at 25 °C for 15 min. The supernatant was measured at 532 nm. The results were determined from a standard curve (0–3000-µM malondialdehyde—MDA) and expressed as the mg MDA/kg of chilled pork (*n* = 3).

#### 2.6.3. Total Plate Count Analysis

Pork samples (25 g) were added to 225 mL of 0.1% peptone water and blended in a Stomacher (IUL-Instruments, Barcelona, Spain) for 2 min (*n* = 3). Subsequent dilutions were prepared using 9 mL of 0.1% peptone water and 1 mL of sample prior to the application of the total plate count technique by pouring on PCA plates and incubated at 37 °C for 24 h [31,32]. The results were expressed as log CFU/g.

#### 2.6.4. Sensory Evaluation

The sensory evaluation was carried out using 50 untrained panelists (male and female, 50:50) following the American Meat Science Association [33] and Vilar et al. [34] recommendations. The ages of the consumers ranged from 19–50 years old. The consumer preferences were evaluated using a 9-point hedonic scale (from 1 = extremely dislikely to 9 = extremely likely), as described by Phimolsiripol et al. [35] and Chokumnoyporn et al. [36]. Consumer sensory panels were done in the sensory normalized testing room at the Chiang Mai University Sensory Research Unit, which conformed to the international standards (ISO) [37]. The analysis was done for 6 samples of fresh, chilled pork, evaluated in two sensory sessions within the same day, which were kept at 0 and 4 °C for up to 8 days of storage. Samples were served on polyethylene trays at 25 °C in a random order. Each sample was coded with a three-digit random number. Water was available for use between samples. The attributes of the unwrapped pork samples were appearance, color, odor, and overall liking.

### 2.7. Statistical Analysis

The statistical analysis was applied following the method of Biffin et al. [38], with a slight modification. The antioxidant properties, including TPC, DPPH IC_50_, ABTS IC_50_, and FRAP, were analyzed and compared to determine the effects of the power and time of the ultrasonication compared to the non-ultrasonicated sample using an analysis of variance (ANOVA) at the 95% confidence level (*p* ≤ 0.05). The experimental design was a factorial in the CRD model using the Statistical Package for the Social Sciences (SPSS version 17.0, SPSS, IBM Corp., Armonk, NY, USA). Mean comparisons were done using Duncan’s post-hoc test at *p* ≤ 0.05. Fixed effects in the full models included the power and time of the ultrasonication (treatments). The relationship or interaction terms between the responses as a function of the power and time of the ultrasonication and optimized optimal conditions were calculated using Design-Expert (Version 6.0.2, Stat-Ease, Inc., Minneapolis, MN, USA). Random terms for all the models included the extraction processing day and replication. Mean values and standard errors of the data were then reported.

For shelf life testing, the physical properties, including the color (L*, a*, and b*); pH; TBARS; and sensory data (appearance, color, odor, and overall liking), were calculated and compared using the ANOVA at the 95% confidence level (*p* ≤ 0.05) using SPSS. The fixed variables for the full models included the concentration of ultrasonicated CE, storage temperature, and storage time treatments. Random terms were grouped according to their relations to the sensory panel (test day, session number, testing order, and panelist). The microbial analysis was regressed to a linear equation showing the kinetic reaction rate (*k*) to delineate the trends of the microbial populations after storage at varied ultrasonicated CE concentration and storage temperature levels, as described by Phimolsiripol et al. [39].

## 3. Results and Discussion

### 3.1. Antioxidant Properties of the Ultrasonicated Extracts

#### 3.1.1. Total Phenolic Compounds (TPC)

**Figure 3:**
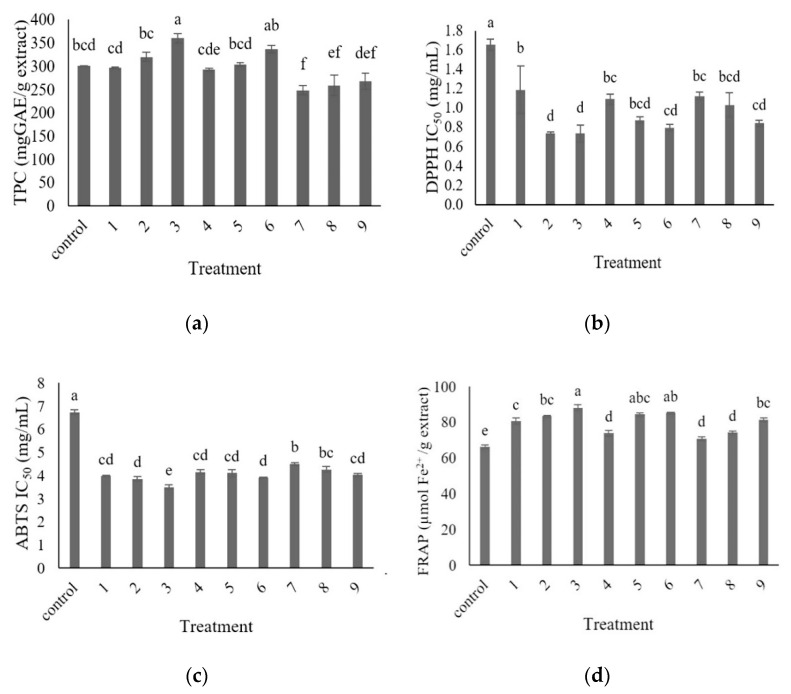
Means and standard errors of the antioxidant properties of the sonicated extracts’ (**a**) Total phenolic compounds (TPC), (**b**) IC_50_ values of the 2,2-Diphenyl-1-picrylhydrazyl (DPPH) radical, (**c**) IC_50_ values of the 2,2′-azino-bis (3-ethylbenzothiazoline-6-sulfonic acid (ABTS) radical, and (**d**) FRAP—ferric-reducing antioxidant power (control = non-sonicated extract, 1 = 60% 10 min, 2 = 60% 25 min, 3 = 60% 40 min, 4 = 80% 10 min, 5 = 80% 25 min, 6 = 80% 40 min, 7 = 100% 10 min, 8 = 100% 25 min, and 9 = 100% 40 min). Different letters indicate significant differences between the treatments (*p* ≤ 0.05).

The TPC of the CE ultrasonicated at 60% power for 40 min significantly increased (*p* ≤ 0.05) when compared to the control, as shown in Figure 3a. Increasing the time of the ultrasonication increased the TPC, which is consistent with previous studies [10]. The results indicated disruption of the plant cell walls by ultrasound waves. The phenolic compounds were released from within the solid matrix. The 100% ultrasonication power resulted in a lower TPC when compared to the control and 60% or 80% ultrasonication power. An increase in the power of the ultrasonication decreased the TPC, presumably due to the destruction of some of the extracted phenolic compounds [40], resulting in a reduction in the TPC when applied with too strong an ultrasonic power.

#### 3.1.2. DPPH and ABTS Radical Scavenging Activity and Ferric-Reducing Antioxidant Power (FRAP)

There was a significant variation in the DPPH IC_50_ values of the ultrasonicated extracts, as shown in Figure 3b. All ultrasonicated extracts showed higher DPPH radical scavenging activities than the non-ultrasonicated extracts (control). The extract with the highest DPPH radical scavenging activity was obtained by ultrasonication at 60% power for 25 and 40 min. Ultrasonication at 60% power for 10 min resulted in the extract with the lowest (*p* ≤ 0.05) DPPH antioxidant capacity. Increasing the time of the ultrasonication led to increasing the DPPH antioxidant activity for 60% power. These results were consistent with an earlier report by Altemimi et al. [41] that showed the degradation of phenolic compounds when using high powers and high temperatures, thereby producing a cavitation bubble collapse.

The ABTS radical scavenging activity was quantified as the reduction in ABTS^+^ radicals and expressed as IC_50_ values (Figure 3c). The extracts with the highest and lowest potentials to inhibit the ABTS radicals were obtained by ultrasonication at 60% power for 40 min and 100% power for 10 min, respectively. The data showed that increasing the time of the ultrasonication increased the ABTS radical scavenging activity for the extracts ultrasonicated at 60% and 100% power. Higher ultrasonication times have been associated with higher flavonoid yields at the same ultrasonic power [10]. A longer extraction time permits more contact time for the cavitation bubbles to rupture more plant cells, in turn increasing the TPC extraction [40]. Therefore, the antioxidant capacities will increase. Ultrasound water baths produce enough cavitation to create shear forces to break the cell walls. Furthermore, ultrasonication increases the diffusion of cell contents into the extraction solution [42].

The FRAP of the extracts are shown in Figure 3d. The extract ultrasonicated at 60% power for 40 min showed the highest (*p* ≤ 0.05) potential to decrease the ferric ions (Fe^3+^), while the lowest potential to decrease the Fe^3+^ ions was observed with the extract ultrasonicated at 100% power for 10 min, suggesting that increasing the time of ultrasonication also increased the FRAP. Compared with the control (non-ultrasonicated sample), there was a significant increase (7–33%, *p* ≤ 0.05) in the FRAP values after ultrasonication. This trend was consistent with Ilghami et al. [43]. It might be due to an increase in the ultrasonic times, which can increase the diffusivity of the solvent into cells and enhance the desorption and solubility of the target compounds from the cells, thereby improving the antioxidant efficacy [44].

### 3.2. Antibacterial Activities of Ultrasonicated Extracts Using MIC and MBC

The antibacterial properties of the ultrasonicated extracts were measured by determining the MIC values (Table 1). The lowest MIC value of all nine extracts able to inhibit the growth of *S. aureus* and *E. coli* was 100-mg/mL. When using 60% power, the extracts inhibited *S. typhimurium* and *P. aeruginosa* at a MIC of 100 mg/mL. When the power was increased, the MIC values against *S. typhimurium* and *P. aeruginosa* were 50 mg/mL. It was evident that increasing the power resulted in increasing the antibacterial activities against *S. typhimurium* and *P. aeruginosa*. The MBC defined the lowest concentration of the extract that could inhibit the tested microorganisms. The MBC values showed the results of an in vitro test in which the fixed concentration of the extracts was being tested against an initially fixed concentration of microorganism [45]. All extracts had an MBC of a 100 mg/mL against *S. aureus* and *E. coli.* When ultrasonicated at 60% power for 10, 25, and 40 min, the MBC values against *S. typhimurium* and *P. aeruginosa* were 100 mg/mL. At 80% and 100% power for 10, 25, and 40 min, the MBC values against *S. typhimurium* and *P. aeruginosa* decreased to 50 mg/mL (Table 1).

A comparison between the ultrasonicated and non-ultrasonicated (control) extracts showed that the antibacterial activities against *S. aureus* and *E. coli* were similar. Whereas the antibacterial activities against *S. typhimurium* and *P. aeruginosa* increased when the ultrasonication power was increased to 80% and 100% for 10, 25, and 40 min (Table 1). Ultrasonic waves cause pressure and cavitation with the disruption of cell walls, so that the components of interest can be released [46] and the extracts are more easily released [47]. The Gram-positive and Gram-negative bacteria showed different sensitivities due to their different cell wall structures. The Gram-negative cell envelope is a thin structure that is covered by an outer membrane. On the other hand, Gram-positive bacteria lack the outer cell membrane, and the cell wall is typically much thicker, with multiple peptidoglycan layers [48]. Due to these distinct differences, it may be easier to inhibit Gram-negative bacteria than Gram-positive bacteria. Therefore, the MBC values of the extracts against *S. typhimurium* and *P. aeruginosa* were lower than the MBC value against *S. aureus*, which is a Gram-positive bacterium. However, the Gram-negative bacteria *E. coli* could tolerate a higher concentration of extracts than the other Gram-negative bacteria, because *E. coli* has the potential to form a dense biofilm around its cells, thus giving them protection against antibacterial agents [49]. As a result, the MBC values of the extracts against *E. coli* were higher than those for the other Gram-negative bacteria. Annatto dye has also been shown to have a greater antibacterial effect on Gram-positive bacteria (lower MIC and MBC) compared with Gram-negative bacteria [50]. These results were probably due to the presence of lipopolysaccharide in the cell wall of Gram-negative bacteria, which can prevent the influx of active compounds into the cytoplasmic membrane of these bacteria [51].

### 3.3. Optimization of Ultrasonication

**Figure 4:**
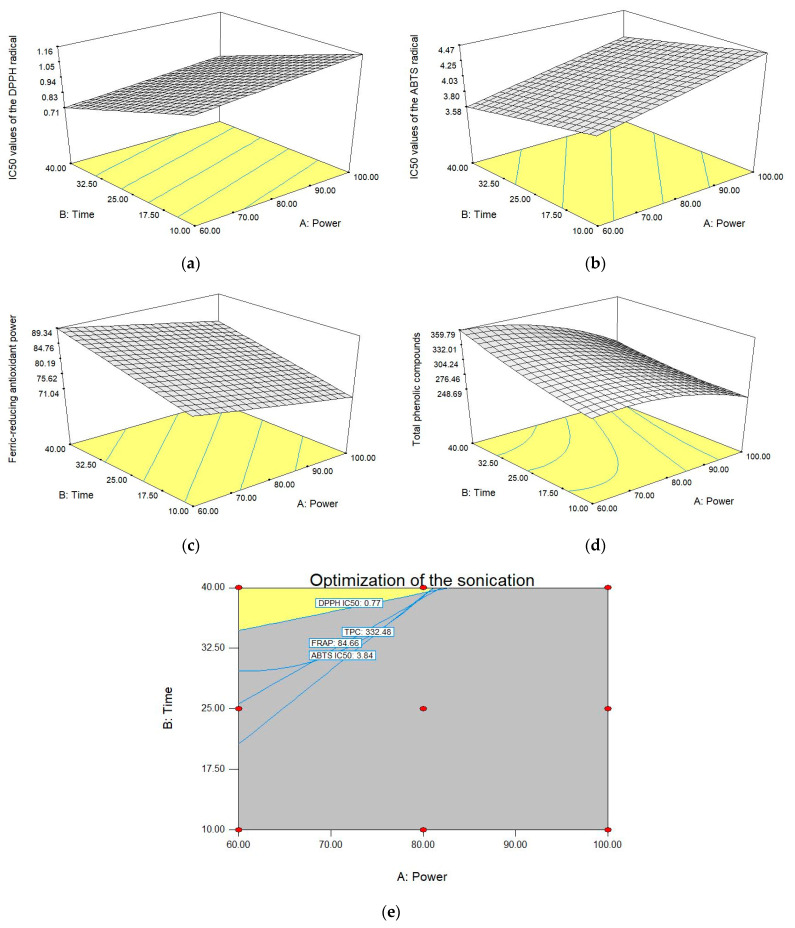
Response surfaces. (**a**) IC_50_ values of the DPPH radical, (**b**) IC_50_ values of the ABTS radical, (**c**) ferric-reducing antioxidant power, (**d**) total phenolic compounds, and (**e**) optimization of the sonication.

The response surface plots (Figure 4) were used to visually observe the relationship between the responses and the various power and times of ultrasonication. The responses studied were TPC, IC_50_ values for the DPPH and ABTS radical scavenging capacities, and FRAP. The response surfaces (Figure 4a–d) were evaluated to predict the optimum power and time of ultrasonication. The optimum condition of ultrasonication was 80% power for 40 min, as shown in Figure 4e. With these conditions, the MIC and MBC values for *S. typhimurium* and *P. aeruginosa* were 50 mg/mL. The content of the TPC was 332-mg GAE/g extract. The IC_50_ values for DPPH and ABTS were 0.77 and 3.84 mg/mL, respectively, and FRAP was 84.7-µmol Fe^2+^/g extract.

### 3.4. Shelf Life Evaluation of Fresh Pork

#### 3.4.1. Color and pH Measurements

**Figure 5:**
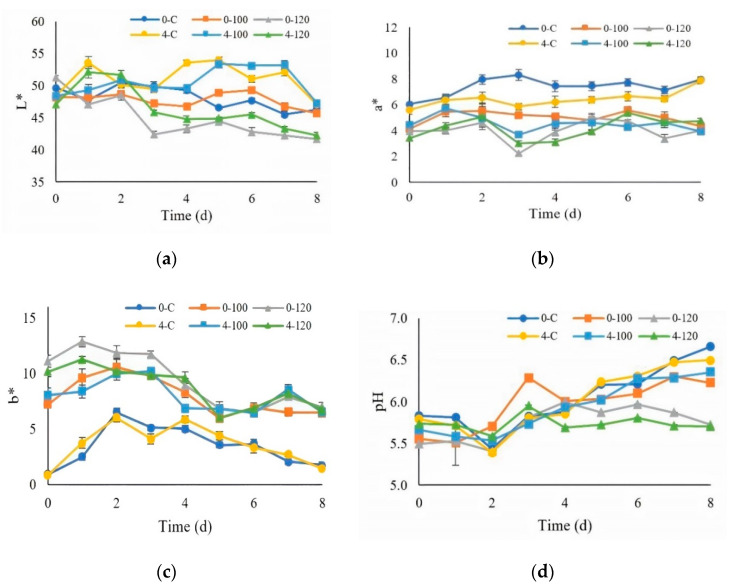
Changes in the means and standard errors of the colors (**a**) Lightness (L*), (**b**) Redness-greenness (a*), and (**c**) Yellowness-blueness (b*) and (**d**) pH when stored at 0 and 4 °C. (0-C = nondipped sample at 0 °C, 0-100 = 100-mg/mL dipped sample at 0 °C, 0-120 = 120-mg/mL dipped sample at 0 °C, 4-C = nondipped sample at 4 °C, 4-100 = 100-mg/mL dipped sample at 4 °C, and 4-120 = 120-mg/mL dipped sample at 4 °C.)

The L* values of all the chilled, fresh pork decreased significantly (*p* ≤ 0.05, Figure 5a) with the increasing storage time. Fresh pork dipped in 120-mg/mL ultrasonicated extract showed lower L* values when compared to fresh pork dipped with 100-mg/mL ultrasonicated extract and the control (nontreated). In addition, the L* value tended to decrease during the increased storage time. Reduction of the L* values reflected pigment materials in the extracts, including chlorophylls [52]. Polyphenol oxidases could oxidize the phenolic compounds to quinones and quinones, which are likely to be condensed to form darker compounds [53]. The control samples (nontreated) showed greater a* values than fresh pork dipped in 100 and 120-mg/mL ultrasonicated extracts at 0–8 day of storage (*p* ≤ 0.05, Figure 5b). Increasing the storage time resulted in an increasing redness (a*) of the control sample at day 8 of storage. The control samples showed lower b* values when compared to fresh pork dipped in the ultrasonicated extract. The values of b* were significantly decreased (*p* ≤ 0.05) with the increasing storage time, as seen in Figure 5c. Increasing the lipid oxidation could lead to a decrease in the a* and b* values [54]. The changes of the pH values of pork when kept at 0 and 4 °C for 8 days are shown in Figure 5d. The pH values of pork significantly increased (*p* ≤ 0.05) with the increased storage time. A similar pH trend was also reported by Lu et al. [31]. The increase of the pH values is associated with bacterial spoilage, related to the action of microbial enzymes, e.g., proteases and lipases, which increase the breakdown of nitrogenous compounds [55]. Changes in the pH may also be related to the spoilage of meat products. The pH usually decreases consequently with the bacterial growth and production of acid from lactic acid bacteria [56].

#### 3.4.2. TBARS Measurement

**Figure 6:**
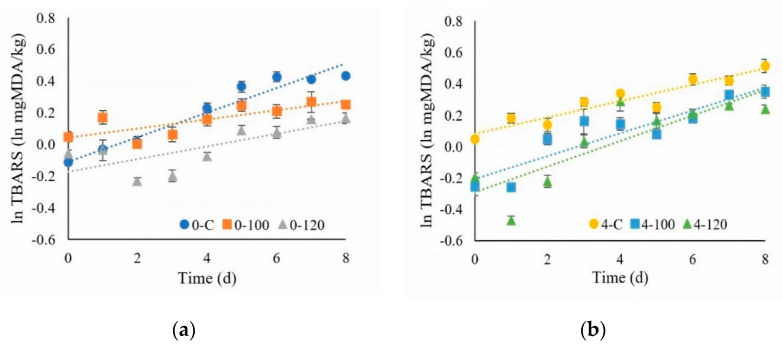
Means and standard errors of the TBARS values of pork sample when stored at (**a**) 0 °C and (**b**) 4 °C. (0-C = nondipped sample at 0 °C, 0-100 = 100-mg/mL dipped sample at 0 °C, 0-120 = 120-mg/mL dipped sample at 0 °C, 4-C = nondipped sample at 4 °C, 4-100 = 100-mg/mL dipped sample at 4 °C, and 4-120 = 120-mg/mL dipped sample at 4 °C.)

The TBARS values of all the chilled, fresh pork increased significantly as the storage time increased (*p* ≤ 0.05, Figure 6a,b). The changes in TBARS in Figure 6a,b show the natural logarithm plot, which indicated a first order-type reaction. The elevation of TBARS with the storage time was observed. Lu et al. [31] mentioned that increasing the storage time resulted in increasing the TBARS values. Fresh pork dipped in 100 and 120-mg/mL ultrasonicated extract showed lower TBARS values compared to the control sample. The results showed that the antioxidative properties of the ultrasonicated extract had the potential to retard the lipid oxidation in pork. Lekjing and Venkatachalam [30] investigated the effects of a chitosan-based coating at 2% incorporated with 1.5% clove oil (CS + CO) on cooked pork sausage samples. They showed that the TBARS values of the CS + CO-treated samples were lower than those of the 2% chitosan-treated samples at 10 and 15 days of storage. Lorenzo et al. [57] also reported that the TBARS values of refrigerated pork patties treated with BHT, green tea extract, seaweed extract, or grape seed extract were lower than in the control samples. The antioxidant properties of the plant extracts could be used to retard the lipid oxidation [58]. Chilled, fresh pork stored at 0 °C had lower TBARS values when compared to fresh pork stored at 4 °C. The lower temperature was effective in decreasing the lipid oxidation [31]

#### 3.4.3. Total Plate Count Analysis

As mentioned by Kim and Jang [32], the total plate count is an important parameter for fresh pork shelf life testing. The total plate counts of chilled, fresh pork with the ultrasonicated extract dipping and nontreated fresh pork (control) are shown in Table 2. The initial microbial loads of the nontreated and treated samples were similar. The total plate counts of all the chilled, fresh pork samples increased linearly with the increased storage time (*p* ≤ 0.05). For storage at 0 °C, the kinetic reaction rate (*k*) values for the control, 100, and 120 mg/mL were 0.698, 0.636, and 0.497 log CFU/g.day, respectively. While, for pork storage at 4 °C, the *k* values for the control, 100, and 120 mg/mL were 0.774, 0.700, and 0.681 log CFU/g.day, respectively. It showed that the 120-mg/mL CE had significantly delayed the growth of the total bacteria. For fresh, chilled pork, the total plate count standard [59] was 6.7 log CFU/g. The total plate counts of the control samples and fresh pork dipped in 100 and 120-mg/mL CE stored at 0 °C reached 6.7 log CFU/g by the 5th, 6th, and 7th days of storage, respectively, with visible signs of spoilage. Meanwhile, the control samples and fresh pork dipped in 100 and 120-mg/mL CE stored at 4 °C had total plate count values above the standard limit on the 4th, 5th, and 6th days of storage, respectively. A lower temperature could extend the storage time, as reported by Akoğlu et al. [60]. It was confirmed that the shelf life of chilled pork was prolonged by dipping with either 100 or 120-mg/mL of CE at a lower temperature (0 °C). This was probably due to the phenolic compounds in the ultrasonicated extracts. The phenolic compounds showed that the antimicrobial activity can increasingly against many Gram-positive bacteria. The Gram-positive bacteria showed a better susceptibility to antimicrobial activity, because the outer membrane of the Gram-negative bacteria represented a reduced absorption of the phenolic compounds and barrier of permeability [61]. They can denature the proteins of microbial cell membranes, leading to inactivation or death [62]. The microbial activities were consistent with the reports for different natural additives and their extracts in sausages [63] and bison meat [64]. The authors de Souza de Azevedo et al. [7] found that nisin can be used as an antimicrobial agent for shelf life extension in pork meat. Ranucci et al. [65] also reported sausage made from pork meat with a mix of *Punica granatum* and *Citrus* spp. extracts. They found that the extracts could extend the shelf life of pork by controlling the microbial growth and oxidation during refrigerated storage at 4 ± 1 °C.

#### 3.4.4. Sensory Evaluation

**Figure 7:**
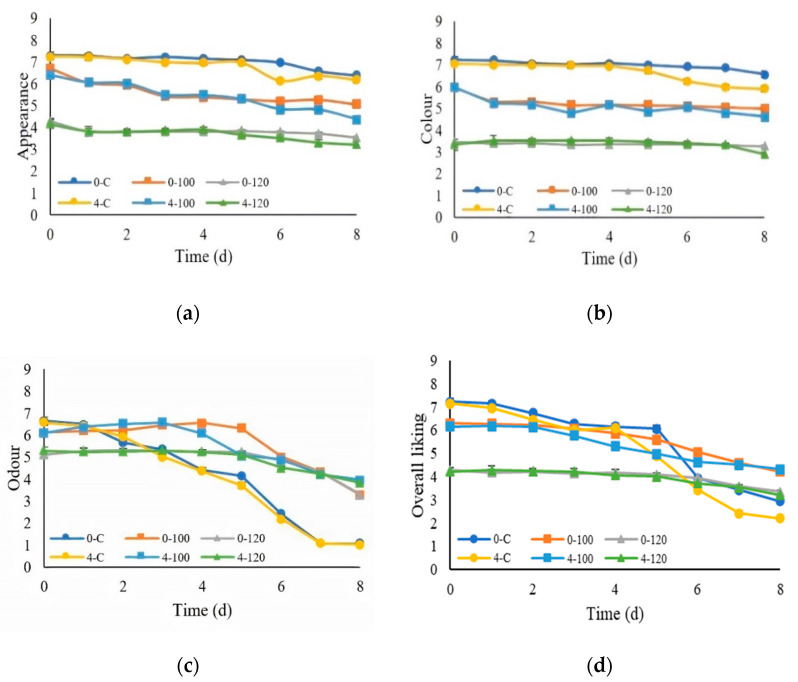
Means and standard errors of the sensory scores from the 9-point hedonic scores of the pork samples: (**a**) appearance, (**b**) color, (**c**) odor, and (**d**) overall liking. (0-C = nondipped sample at 0 °C, 0-100 = 100-mg/mL dipped sample at 0 °C, 0-120 = 120-mg/mL dipped sample at 0 °C, 4-C = nondipped sample at 4 °C, 4-100 = 100-mg/mL dipped sample at 4 °C, and 4-120 = 120-mg/mL dipped sample at 4 °C.)

The sensory evaluation showed that the scores for the appearance, color, odor, and overall liking from all the chilled pork samples showed significant decreases (*p* ≤ 0.05) with the increasing storage time, as shown in Figure 7. The scores for the appearance and color from the control were higher than fresh pork dipped in the ultrasonicated extracts. The result showed that the fresh pork dipped in 120-mg/mL at 0 and 4 °C had lower consumer acceptance (<5 hedonic score) due to too strong a greenish appearance and color, affected by too much CE extract. The control samples at 0 and 4 °C had a spoiled odor after a storage period of 4–8 days, which resulted in lower scores of the overall liking. The consumers preferred fresh pork dipped in ultrasonicated extracts that received higher odor and overall liking scores than the control samples. This was probably due to the antimicrobial effect of the CE, as confirmed by the increased microbial population found in the experiments reported in Section 3.4.3. In addition, Ramírez-Rojo et al. [66] showed that pork patties treated with an ethanol extract of Mesquite leaves could increase the shelf life with acceptable sensory properties. Due to the sensory acceptance limitations of 120-mg/mL CE, 100-mg/mL CE should be selected to apply for shelf life extensions instead of 120-mg/mL CE with better acceptance by consumers.

## 4. Conclusions

Increasing the time of ultrasonication resulted in increasing the antioxidant activities (DPPH, ABTS, and FRAP) of the CE extracts. Increasing the ultrasonication power increased the capacity of the extracts to inhibit the growth of *S. typhimurium* and *P. aeruginosa*. The optimum ultrasonication condition was determined as ultrasonication at 80% power for 40 min. The fresh pork dipped in 100-mg/mL ultrasonicated extract could be kept for 7 days at 0 °C when compared to the control, which could be kept for only 5 days. The CE extracts by ultrasonication showed greater antioxidant and antimicrobial properties for improving the shelf life of fresh pork. In addition, the sensory evaluation of the fresh pork dipped in 100-mg/mL CE at 0 °C was also acceptable to consumers. Accordingly, these can be safety applied by the food industry to enhance the quality and extend the shelf life of meat products. More work is required to investigate the kinetic study of extraction using ultrasonication and the flavor profile of ultrasonicated CE alone and its impact on treated foods. More mechanisms of the shelf life studies of CE extracts are also needed.

## Figures and Tables

**Table 1:** Minimum inhibitory concentrations (MIC) and minimum bactericidal concentrations (MBC) of the ultrasonicated extracts.

Power (%)	Time (min)	MIC (mg/mL)				MBC (mg/mL)			
		*S. aureus*	*E. coli*	*S. typhimurium*	*P. aeruginosa*	*S. aureus*	*E. coli*	*S. typhimurium*	*P. aeruginosa*
60	10	100	100	100	100	100	100	100	100
60	25	100	100	100	100	100	100	100	100
60	40	100	100	100	100	100	100	100	100
80	10	100	100	50	50	100	100	50	50
80	25	100	100	50	50	100	100	50	50
80	40	100	100	50	50	100	100	50	50
100	10	100	100	50	50	100	100	50	50
100	25	100	100	50	50	100	100	50	50
100	40	100	100	50	50	100	100	50	50
Control (Non-ultrasonicated extract)		100	100	100	100	100	100	100	100

**Table 2:** Total plate count (log CFU/g) of the pork samples dipped with 100 and 120-mg/mL CE in comparison with the undipped samples (control) during storage at 0 and 4 °C.

Storage Day	0 °C			4 °C		
	Control	100 mg/mL	120 mg/mL	Control	100 mg/mL	120 mg/mL
0	3.33 ± 0.11 ^aG^	3.21 ± 0.26 ^aG^	2.99 ± 0.07 ^aE^	3.09 ± 0.39 ^aF^	3.05 ± 0.18 ^aG^	3.17 ± 0.13 ^aG^
1	3.67 ± 0.06 ^bF^	2.79 ± 0.01 ^dH^	3.08 ± 0.15 ^cE^	3.64 ± 0.03 ^bE^	4.09 ± 0.04 ^aE^	2.74 ± 0.09 ^dH^
2	3.21 ± 0.08 ^cG^	3.57 ± 0.04 ^bF^	2.88 ± 0.11 ^dEF^	4.03 ± 0.01 ^aD^	3.66 ± 0.06 ^bF^	3.34 ± 0.06 ^cG^
3	4.41 ± 0.02 ^bE^	3.86 ± 0.07 ^dE^	2.71 ± 0.03 ^fF^	5.35 ± 0.10 ^aC^	4.27 ± 0.05 ^cE^	3.58 ± 0.03 ^eF^
4	4.36 ± 0.03 ^bE^	4.95 ± 0.02 ^aC^	4.07 ± 0.23 ^cD^	5.15 ± 0.06 ^aC^	4.10 ± 0.10 ^bcE^	3.83 ± 0.02 ^cE^
5	5.93 ± 0.22 ^cD^	4.68 ± 0.08 ^eD^	4.05 ± 0.04 ^fD^	7.21 ± 0.03 ^aB^	6.30 ± 0.03 ^bD^	5.36 ± 0.07 ^dD^
6	6.70 ± 0.08 ^cC^	6.13 ± 0.01 ^eB^	5.21 ± 0.03 ^fC^	8.48 ± 0.01 ^aA^	7.08 ± 0.03 ^bC^	6.24 ± 0.02 ^dC^
7	8.04 ± 0.02 ^bB^	6.21 ± 0.01 ^eB^	6.44 ± 0.05 ^dB^	8.48 ± 0.01 ^aA^	7.88 ± 0.09 ^bB^	7.42 ± 0.16 ^cB^
8	8.48 ± 0.01 ^aA^	7.15 ± 0.10 ^bA^	7.22 ± 0.06 ^bA^	8.48 ± 0.01 ^aA^	8.48 ± 0.01 ^aA^	8.48 ± 0.01 ^aA^

Different lowercase letters (a–f) indicate significant differences between treatments (*p* ≤ 0.05), and different uppercase letters (A–H) indicate significant differences between the times (*p* ≤ 0.05). A green color indicates the end of the shelf life (day). A red color indicates the measured values of the rejected samples after the end of the shelf life.

## Data Availability

Not applicable.
